# Downregulation of lncRNA CCDC26 contributes to imatinib resistance in human gastrointestinal stromal tumors through IGF-1R upregulation

**DOI:** 10.1590/1414-431X20198399

**Published:** 2019-06-03

**Authors:** Jingyi Yan, Didi Chen, Xiaolei Chen, Xuecheng Sun, Qiantong Dong, Changyuan Hu, Feng Zhou, Wei Chen

**Affiliations:** 1 Department of Gastroenterology and General Surgery, The First Affiliated Hospital of Wenzhou Medical University, Wenzhou, Zhejiang Province, China; 2 Department of Radiotherapy and Medical Oncology, The First Affiliated Hospital of Wenzhou Medical University, Wenzhou, Zhejiang Province, China; 3 Department of Gastroenterology and Hepatology, The First Affiliated Hospital of Wenzhou Medical University, Wenzhou, Zhejiang Province, China; 4 Tongde Hospital of Zhejiang Province, Hangzhou, Zhejiang Province, China

**Keywords:** Long non-coding RNA CCDC26, Gastrointestinal stromal tumors, GIST, IGF-1R, Imatinib resistance

## Abstract

Imatinib is the first line of therapy for patients with metastatic or gastrointestinal stromal tumors (GIST). However, drug resistance limits the long-term effect of imatinib. Long non-coding RNAs (lncRNAs) are emerging as key players in regulating drug resistance in cancer. In this study, we investigated the association between lncRNA CCDC26 and IGF-1R in GIST and their involvement in drug resistance. Considering the key role of lncRNAs in drug resistance in cancer, we hypothesized that IGF-1R is regulated by lncRNAs. The expression of a series of reported drug resistance-related lncRNAs, including CCDC26, ARF, H19, NBR2, NEAT1, and HOTAIR, in GIST cells treated with imatinib H19 was examined at various time-points by qRT-PCR. Based on our results and published literature, CCDC26, a strongly down-regulated lncRNA following imatinib treatment, was chosen as our research target. GIST cells with high expression of CCDC26 were sensitive to imatinib treatment while knockdown of CCDC26 significantly increased the resistance to imatinib. Furthermore, we found that CCDC26 interacted with c-KIT by RNA pull down, and that CCDC26 knockdown up-regulated the expression of IGF-1R. Moreover, IGF-1R inhibition reversed CCDC26 knockdown-mediated imatinib resistance in GIST. These results indicated that treatments targeting CCDC26-IGF-1R axis would be useful in increasing sensitivity to imatinib in GIST.

## Introduction

Imatinib is the only approved first-line drug for gastrointestinal stromal tumor (GIST) patients, especially for patients with advanced or metastatic tumors (1,2). Imatinib has significantly improved the prognosis of end-stage patients. Disease control ranges from 70 to 85%, median progression-free survival is 29 months, and median overall survival is 57 months (3–5). GIST patients treated with imatinib are confronted with primary and secondary drug resistance. The drug resistance limits the long-term curative effect of imatinib (6–8). However, the underlying molecular mechanisms of imatinib resistance in GIST have not been fully elucidated.

Long non-coding (lnc) RNAs are non-protein coding RNAs functionally defined as transcripts >200 nucleotides in length. These account for at least 80% of transcripts in the genome (9,10). Previous studies demonstrated that lncRNAs are involved in the regulation of drug resistance in many cancers (11). Some well-studied lncRNAs, including H19, NBR2, ARF, HOTAIR (HOX transcript antisense RNA), and NEAT1, have been shown to act as oncogenes or tumor suppressor genes, which are also correlated with drug resistance (12–16). For example, H19 regulates cisplatin resistance in human lung adenocarcinoma cells and NBR2-GLUT1 axis regulates cancer cell sensitivity to biguanides (12,13 [Bibr B14]). Furthermore, HOTAIR inhibition results in higher sensitivity to imatinib by regulating MRP expression (15). Knockdown of lncRNA NEAT1 could promote imatinib-induced apoptosis by regulating c-Myc (16).

lncRNA CCDC26, located on chromosome 8q24.21, was first reported as a retinoic acid-dependent modulator of myeloid differentiation; it is also called RAM (17). Previous studies have concentrated on CCDC26 polymorphism related to glioma risk (18,19). Very few reports characterize the function of CCDC26 in other cancers. In acute myeloid leukemia, CCDC26 controls growth of myeloid leukemia cells through the regulation of KIT (20). In pancreatic cancer, CCDC26 was labeled as a novel oncogene and is responsible for the growth and apoptosis of cancer cells by regulating PCNA and Bcl2 expression (20,21). However, there are no reports on the biological function of CCDC26 in imatinib resistance in GIST.

In this study, we investigated the role of CCDC26 in the sensitivity of GIST cells to imatinib and revealed a potential mechanism.

## Material and Methods

### Cell culture and transfection

Human GIST cell lines GIST-882 and GIST-T1 were purchased from the Chinese Academy of Science Cell Bank (China), and maintained in RPMI medium (Gibco, USA) supplemented with 10% fetal bovine serum (Gibco) and 1% penicillin/streptomycin (Gibco) at 37°C and 5% CO_2_. IGF-1R and CCDC26 siRNA were synthesized by GenePharma (China) using the following sequences: CCDC26 siRNA: sense: 5′-CCUACCACACAACCACUUUTT-3′, antisense: 5′-AAAGUGGUUGUGUGGUAGGTT-3′; IGF-1R siRNA: sense: 5′-CCAAGCUAAACCGGCUAAATT-3′, antisense: 5′-UUUAGCCGGUUUAGCUUGGTT-3′. The transfection was conducted using lipofectamine 2000 (Invitrogen, USA) according to the manufacturer’s instructions.

### CCK-8 assay

A colorimetric assay using CCK-8 (Dojindo Laboratories, Japan) was used to assess cell viability. Briefly, 5000 cells from each group were plated in 96-well plates in 200 μL of RPMI 1640 culture medium containing 10% FBS. Then, the cells were cultured with different treatments for 48 h. Ten microliters of CCK-8 in 100 μL medium was added to each well and incubated for 2 h according to the manufacturer’s instructions. An MRX II microplate reader (Dynex, USA) was used to measure the absorbance value at 450 nm.

### EDU assay

Cell proliferation of GIST cells was assayed using the Click-iTEdU Imaging kit (Invitrogen) according to the manufacturer’s protocol. Hoechst^®^ 33342 solution was used to stain nuclei. EDU-stained cells were mounted and imaged by fluorescence microscopy.

### Flow cytometric analysis of apoptosis

Apoptosis of GIST cells was detected using annexin V-FITC Apoptosis Detection kit (Abcam, USA) according to the manufacturer’s protocol. Apoptosis rates were measured using a flow cytometer (LSRII, BD Biosciences, USA). Cells in the Q2 and Q3 quadrants were considering as apoptotic.

### Western blot

Treated GIST cells were washed in phosphate-buffered saline (PBS) twice before proteins were extracted and quantified using a BCA kit (Thermo scientific, USA). Then, 40 μg of protein in each group was separated on a SDS/PAGE gel, transferred onto a PVDF membrane, and subjected to immunoblot analysis. The primary antibodies against IGF-1R (diluted: 1:1000, ab39398) and GAPDH and the corresponding secondary antibodies (diluted: 1:2000, ab7090) were obtained from Abcam.

### Quantitative real-time PCR

Total RNA was extracted using the TRIzol reagent (Invitrogen) according to manufacturer’s instructions. Reverse transcription was performed to obtain the first strand cDNA using the PrimeScript^®^ RT reagent kit (Takara, China). The relative expressions of CCDC26, ARF, H19, NBR2, NEAT1, and HOTAIR were normalized to β-actin. The relative expression of IGF-1R was normalized to the internal reference GAPDH. All qRT-PCR reactions were performed using an ABI Prism 7500 (Applied Biosystems, USA). The results were analyzed using the 2^-ΔΔCt^ method. CCDC26: forward primer: 5′-GGAUAUGUCAAUCUCACAATT-3′; reverse primer: 5′-UUGUGAGAUUGACAUAUCCTT-3′. Negative siRNA forward primer: 5′-UUCUCCGAACGUGUCACGUTT-3′; reverse primer: 5′-ACGUGACACGUUCGGAGAATT-3′. IGF1-R siRNA forward primer: 5′-AGTGGAGAAATCTGCGGGC-3′; reverse primer: 5′-ACTCGGTAATGACCGTGAGC-3′. GAPDH forward primer: 5′-UGACCUCAACUACAUGGUUTT-3′; reverse primer: 5′-AACCAUGUAGUUGAGGUCATT-3′.

### RNA pull-down assay

RNA pull-down was performed using a Magnetic RNA-Protein Pull-Down kit (Pierce, USA) in accordance with the manufacturer’s instructions. Briefly, CCDC26 RNA or anti-sense CCDC26 RNA was labeled with magnetic beads. Proteins extracted from GIST cells were mixed with magnetic beads labeled RNA. The associated proteins were resolved by SDS-PAGE.

### Statistical analysis

All data are reported as means±SE. Statistical analyses were performed with Student’s *t*-test (parametric) or Mann-Whitney (non-parametric) test, and one-way analysis of variance followed by Tukey’s *post hoc* test using SPSS 18.0 software (IBM, USA). P<0.05 was considered significant.

## Results

### IGF-1R knockdown sensitized GIST cells to imatinib

**Figure 1:**
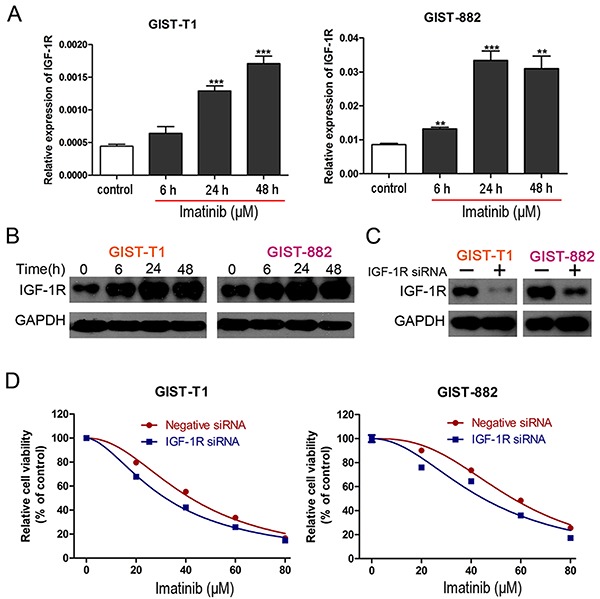
A, mRNA level of IGF-1R in GIST-T1 and GIST-882 cells treated with imatinib (GIST-T1, 41.97 μM; GIST-882, 56.90 μM) at different time-points by real time PCR. Data are reported as means±SE. **P<0.01, ***P<0.001 *vs* control (one-way analysis of variance followed by Tukey’s *post hoc* test). **B**, Protein level of IGF-1R in GIST-T1 and GIST-882 cells treated with imatinib at different time-points by western blot. **C**, The interfering efficiency of IGF-1R confirmed by western blot. **D**, Cell viability of GIST-T1 and GIST-882 cells with or without IGF-1R knockdown in the presence of different concentrations of imatinib.

IGF-1R was previously shown to be upregulated in wild-type GISTs, which were insensitive to imatinib (22). It was also reported that IGF-1R is involved in imatinib resistance in GIST (23). In this study, the mRNA and protein level of IGF-1R was reduced in a time-dependent manner in GIST cells cultured with imatinib (Figure 1A and B). To further explore the role of IGF-1R on imatinib resistance in GIST, IGF-1R siRNA was employed. The interfering efficiency was confirmed by western blot (Figure 1C). Following transfections of IGF-1R siRNA or negative siRNA, cells were exposed to different concentrations of imatinib for 48 h. Then, cell viability was assessed by CCK8 assay. Down-regulation of IGF-1R resulted in decreased cell viability following imatinib treatment in GIST cells (Figure 1D). These results indicated that overexpression of IGF-1R, which was observed in imatinib-treated cells, was responsible for the development of imatinib resistance in GIST cells.

### Expression of lncRNAs in GIST cells treated with imatinib

**Figure 2:**
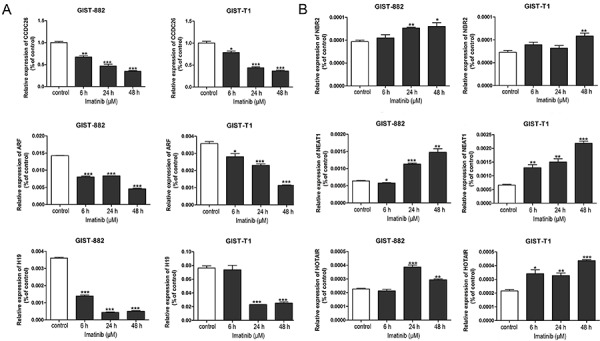
GIST-T1 and GIST-882 cells were treated with imatinib (GIST-T1, 41.97 μM; GIST-882, 56.90 μM) and RNA extracted for real time PCR. **A**, Expression of CCDC26, ARF, and H19 assayed by real time PCR. **B**, Expression of NBR2, NEAT1, and HOTAIR assayed by real time PCR. U6 was used as the internal control. Data are reported as means±SE. *P<0.05, **P<0.01, ***P<0.001 *vs* control (one-way analysis of variance followed by Tukey’s *post hoc* test).

The results of qPCR showed that the expression of CCDC26 decreased in a time-dependent manner after culture with imatinib in both GIST-T1 and GIST-882 cells, and expression of NEAT1 increased in a time-dependent manner. However, the expression of ARF, H19, NBR2, and HOTAIR did not change significantly in the two GIST cell lines (Figure 2A and B). Considering the opposing regulation of CCDC26 and IGF-1R in GIST cells treated with imatinib, it is possible that CCDC26 could increase the sensitivity of GIST cells to imatinib.

### Knockdown of CCDC26 was sufficient for imatinib resistance in GIST cells

**Figure 3:**
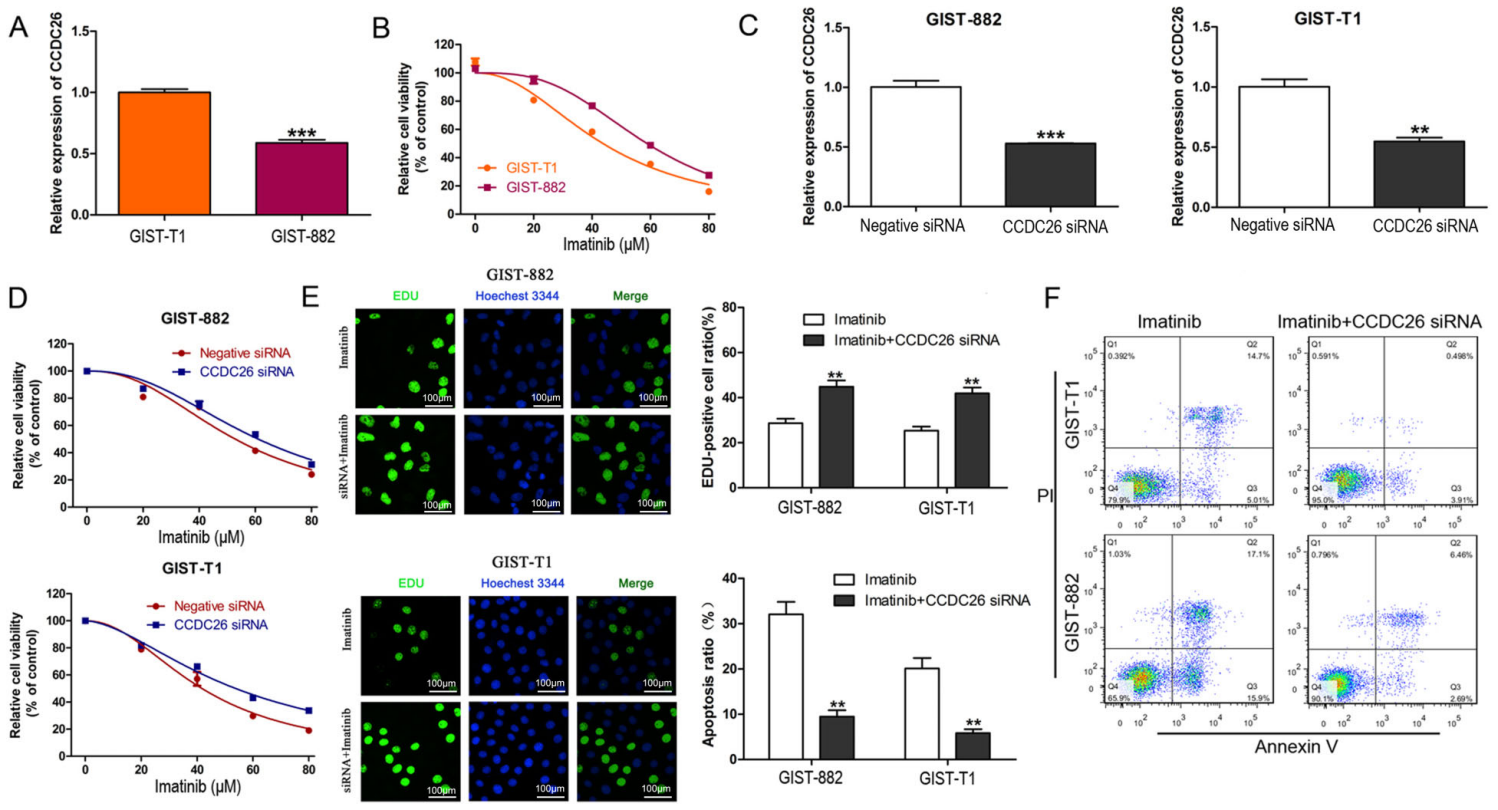
Knockdown of CCDC26 induced imatinib resistance in gastrointestinal stromal tumors (GIST) cells. **A**, Expression of CCDC26 in GIST-T1 and GIST-882 cells by real time PCR. ***P<0.001 *vs* GIST-T1. **B**, Cell viability of GIST-T1 and GIST-882 cells in the presence of different concentrations of imatinib by CCK-8 assay. **C**, The interfering efficiency validation of CCDC26 siRNA by real time PCR. **P<0.01, ***P<0.001 *vs* Negative siRNA (Student’s *t*-test). **D**, CCK-8 assays were performed to determine the cell viability of GIST-T1 and GIST-882 cells treated with CCDC26 siRNA or control siRNA in the presence of different concentrations of imatinib. **E**, EDU assay was employed to examine cell proliferation in GIST-T1 and GIST-882 cells cultured with imatinib (GIST-T1, 41.97 μM; GIST-882, 56.90 μM) and treated with CCDC26 or control siRNA (×200 magnification; bars: 100 μM). The number of EDU positive cells was counted. **P<0.01 *vs* imatinib (Student’s *t*-test (parametric); Mann-Whitney test (non-parametric)). **F**, Flow cytometric analysis of apoptotic GIST-T1 and GIST-882 cells transfected with control or CCDC26 siRNA and treated with imatinib for 48 h, **P<0.01 *vs* imatinib (Student’s *t*-test (parametric); Mann-Whitney test (non-parametric)).

GIST-882 cells expressed lower CCDC26 compared to GIST-T1 cells (Figure 3A). However, the CCK-8 assay indicated that GIST-882 cells were more resistant to imatinib than GIST-T1 cells (Figure 3B). Furthermore, CCDC26 knockdown significantly increased GIST cell viability in the presence of imatinib (Figure 3D). The interfering efficiency of CCDC26 siRNA was confirmed by qPCR (Figure 3C). Cell proliferation was also increased in si-CCDC26-transfected GIST cells treated with imatinib compared to si-NC-transfected controls (Figure 3E). Flow cytometry demonstrated that CCDC26 knockdown gradually decreased the imatinib-induced apoptosis of GIST cell (Figure 3F). These results revealed that CCDC26 knockdown enhanced the imatinib resistance of GIST cells.

### CCDC26 knockdown induced imatinib resistance by regulating IGF-1R expression in GIST cells.

**Figure 4:**
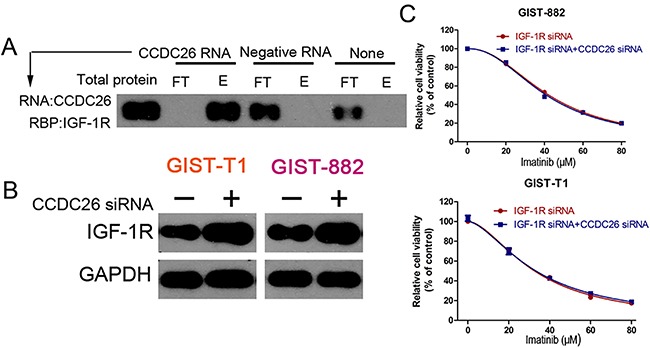
CCDC26 pull down of IGF-1R and its regulation and expression. **A**, Expression levels of IGF-1R were assayed in CCDC26 RNA group (target), control RNA group (unrelated), and blank group (none) following RNA pull down (FT: flow-through T: eluate). **B**, Expression of IGF-1R in GIST-T1 and GIST-882 cells with the transfection of CCDC26 siRNA or control RNA. **C**, Cell viability of GIST-T1 and GIST-882 cells transfected with IGF-1R siRNA with control siRNA or CCDC26 siRNA, and IGF-1R siRNA treated with different concentrations of imatinib for 48 h.

Considering the opposing expression and role of CCDC26 and IGF-1R in GIST cells, we hypothesized that CCDC26 could interact with IGF-1R. To support this, we performed an RNA pull down experiment. Our results demonstrated that CCDC26 RNA could pull down IGF-1R protein (Figure 4A). Furthermore, we found that CCDC26 knockdown up-regulated IGF-1R (Figure 4B). To further investigate the relationship between CCDC26 and IGF-1R, we tested cell viability in GIST cells after transfection with CCDC26 siRNA or negative siRNA, which were pretreated with IGF-1R siRNA. The result revealed that IGF-1R knockdown abolished CCDC26 inhibition-mediated imatinib resistance (Figure 4C). These results indicated that CCDC26 knockdown induced imatinib resistance in GIST cells through IGF-1R interaction.

## Discussion

GIST is the most common sarcoma of the gastrointestinal tract (24,25), and it is defined as a potentially malignant gastroenteric tumor, causing impairments to patients (26,27). In recent years, anti-cancer drug resistance remains one of the most significant challenges to successful treatment of cancer (28). Although the mechanism of drug resistance has not been fully elucidated, many studies have demonstrated that lncRNAs might be a key regulator in drug resistance and showed potential in clinical application (29,30). Furthermore, up to now, there are few reports on the functions of lncRNAs in GIST progression and imatinib resistance. In this study, we explored the role and mechanism of lncRNA CCDC26 in imatinib resistance in GIST cells and demonstrated for the first time that CCDC26 enhanced imatinib sensitivity by downregulating IGF-1R expression.

It had been revealed that lncRNAs might play an important function in cancer by regulating a series of critical biological functions, including cell proliferation, apoptosis, and drug resistance (9,31). For example, lncRNA CPS1-IT1 could significantly reduce cell proliferation, migration, and invasion capacities, and accelerate cell apoptosis by suppressing epithelial-mesenchymal transition (32). lncUCA1 overexpression and miR-27b inhibition increased ADR, DDP, and 5-FU resistance and reduced ADR-induced cell apoptosis in gastric cancer cells (33). The functional roles of lncRNAs in GIST indicate its potential employment as biomarkers and therapeutic targets in GIST (34). Upregulation of HOTAIR is associated with GIST malignancy (35). In the present study, we firstly screened six lncRNAs involved in drug resistance in previous studies in GIST cells following imatinib treatment. We found that the level of CCDC26, which was rarely studied, was reduced in a time-dependent manner in two GIST cell lines cultured with imatinib. To obtain insight on the role of CCDC26 on imatinib resistance of GIST, we examined cell viability and proliferation, and apoptosis activity demonstrating that CCDC26 knockdown induced GIST imatinib resistance and decreased the imatinib-induced apoptosis of GIST compared with the imatinib alone treatment group. These results indicated CCDC26 could regulate imatinib resistance of GIST, which could be a target to reversing imatinib resistance.

IGF-1R is a receptor tyrosine kinase, binds to its ligand IGF1 and IGF2, and regulates Ras-Raf-ERK-MAPK and PI3K-AKT-mTOR signaling pathways (36). IGF-1R is implicated in many cancers and plays an essential role in cancer development and progression (37). In a previous study, IGF-1R was upregulated in a subset of GISTs and over-expressed in wild type and pediatric GISTs (22). IGF-1R inhibitors were shown to have a potential for combined approaches in patients with pediatric GIST and adult wild type GIST (38). In our study, we also demonstrated IGF-1R upregulation in GIST cells following imatinib treatment. Knockdown of IGF-1R significantly improved sensitivity to imatinib in GIST cells. Though the definite role of IGF-1R in imatinib resistance in GIST has not yet been fully elucidated, it has been reported that some lncRNAs could regulate IGF-1R (39,40). However, there was no report about CCDC26 and IGF-1R. In this study, we first revealed that CCDC26 interacted with IGF-1R, which could pull down IGF-1R protein, and CCDC26 knockdown up-regulated IGF-1R. Moreover, we demonstrated that IGF-1R knockdown reversed CCDC26 inhibition-mediated imatinib resistance. These results indicated that IGF-1R was a modulator of CCDC26 mediated sensitivity alteration. Therefore, we demonstrated a CCDC26/IGF-1R axis in the regulation of imatinib resistance in GIST. However, the underlying mechanism linking CCDC26 or IGF-1R was not addressed and should be revealed in the future.

In conclusion, our study demonstrated that CCDC26 could regulate imatinib resistance in GIST. We further verified that CCDC26 interacted with IGF-1R. Our results certified that CCDC26 improved imatinib chemosensitivity in GIST, and indicated that CCDC26 may be used as a therapeutic target to reverse the imatinib resistance of GIST patients.
